# Supplementary material for: Heat adaptation of phage T7 under an extended genetic code
Source: Virus Evol. 2021 Dec 1;7(2):veab100. doi: 10.1093/ve/veab100 (PMC8923235; doi:10.1093/ve/veab100)
Supplement: veab100_Supp [file veab100_supp.zip › Sequencing_Analysis.zip!/Sequencing_Analysis/A3/evidence/RA_42.html]

BRESEQ :: Evidence


| Read alignment evidence... | | | | | | | | | | | |
| --- | --- | --- | --- | --- | --- | --- | --- | --- | --- | --- | --- |
|  | seq id | position | | ref | new | freq | score (cons/poly) | reads | annotation | genes | product |
| \* | NC\_001604.1 | 29,691 | 0 | A | G | 57.8% | 795.6 / inf | 1484 | intergenic (–/–) | *–/–* | –/– |
| Reads supporting (aligned to +/- strand):  **ref** base A (305/317);  **new** base G (422/430);  **total** (733/751) | | | | | | | | | | | |
| Fisher's exact test for biased strand distribution *p*-value = 8.74e-01 | | | | | | | | | | | |
| Kolmogorov-Smirnov test that lower quality scores support variant *p*-value = 1.22e-02 | | | | | | | | | | | |

Only 100 of 1499 total aligned reads displayed.  

`AGGAACAAGTTCAGAATCAGATGAACGCATGGACGAAAGCTCAGGCCAAGGCTCTGGACGATTCCATGAAGTCAATGAACAAACTTGACGTAATCGACAAGCAATTCCAGAAGCGAATCAACGGTGAGTGGGTCTCAACGGATTTTAAGGATATGCCAGTCAACGAGAACACTGGTGAGTTCAAGCATAGCGATATGGTTAACTACGCCAATAAGAAGCTCGCTGAGATTGACAGTATGGACATTCCAGACGGTGCCAAGGATGCTATGAAGTTGAAGTACCTTCAAGCGGACTCTAAGGACGGAGCATTCCGTACAGCCATCGGAACCATGGTCACTGACGCTGGTCAAGAGTGGTCTGCCGCTGTGATTAACGGTAAGTTACCAGAACGAACCCCAGCTATGGATGCTCTGCGCAGAATCCGCAATGCTGACCCTCAGTTGATTGCTGCGCTATACCCAGACCAAGCTGAGCTATTCCTGACGATGGACATGATGGACAAGCAGGGTATTGACCCTCAGGTTATTCTTGATGCCGACCGACTGACTGTTAAGCGGTCCAAAGAGCAACGCTTTGAGGATGATAAAGCATTCGAGTCT  >  NC_001604.1/29391‑29989`  

                                                                                                                                                                                                                                                                                                            `|`                                                                                                                                                                                                                                                                                                            
 `ggAATAAGTTCAGAATCAGATGAACGCATGGACGAAAGCTCAGGCCAAGGCTCTGGACGATTCCATGAAGTCAATGAACAAACTTGACGTAATCGACAAGCAATTCCAGAAGCGAATCAACGGTGAGTGGGTCTCAACGGATTTTAAGGATATGCCAGTCAACGAGAACACTGGTGAGTTAAAGCATAGCGATATGGTTAACTACGCCAATAAGAAGCTCGCTGAGATTGATAGTATGGACATTCCAGACGGTGCCAAGGATGCTATGAAGTTGAAGAACCTTCAAGCGGACTCTAAGGag                                                                                                                                                                                                                                                                                                           >  1:137630/1‑300 (MQ=255)`  
        `gTTCAGAATCAGATGAACGCATGGACGAAAGCTCAGGCCAAGGCTCTGGACGATTCCATGAAGTCAATGAACAAACTTGACGTAATCGACAAGCAATTCCAGAAGCGAATCAACGGTGAGTGTGTCTCAACGGATTTTAAGGATATGCCAGTCAACGAGAACACTGGTGAGTTCAAGCATAGCGATATGGTTAACTACGCCAATAAGAAGCTCGCTGAGATTGACAGTATGGACATTCCAGACGGTGCCAAGGATGCTATGAAGTTGAAGTACCTTCAAGCGGACTCTAAGGACGGAGAAt                                                                                                                                                                                                                                                                                                    >  1:154463/1‑301 (MQ=255)`  
           `cAGAATCAGATGAACGCATGGACGAAAGCTCAGGCCAAGGCTCTGGACGATTCCATGAAGTCAATGAACAAACTTGACGTAATCGACAAGCAATTCCAGAAGCGAATCAACGGTGAGTGGGTCTCAACGGATTTTAAGGATATGCCAGTCAACGAGAACACTGGTGAGTTCAAGCATAGCGATATGGTTAACTACGCCAATAAGAAGCTCGCTGAGATTGACAGTATGGACATTCCAGACGGTGCCAAGGATGCTATGAAGTTGAAGTACCTTCAAGCGGACTCTAAGGGCGGAGCATTcc                                                                                                                                                                                                                                                                                                 <  1:204165/301‑1 (MQ=255)`  
              `aaTCAGATGAACGCATGGACGAAAGCTCAGGCCAAGGCTCTGGACGATTCCATGAAGTCAATGAACAAACTTGACGTAATCGACAAGCAATTCTAGAAGCGAATCAACGGTGAGTGGGTCTCAACAGATTTTAAGGATATGCCAGTCAACGAGAACACTGGTGAGTTCAAGCATAGCGATATGGTTAACTACGCCAATAAGAAGCTCGCTGAGATTGACAGTATGGACATTCCAGACGGTGCCAAGGATGCTATGAAGTTGAAGTACCTTCAAGCGGACTCTAAGGACGGAGCATTCCGTa                                                                                                                                                                                                                                                                                              >  1:38588/1‑301 (MQ=255)`  
               `aTCAGATGAACGCATGGACGAAAGCTCAGGCCAAGGCTCTGGACGATTCCATTAAGTCAATGAACAAACTTGACGTAATCGACAAGCAATTCCAGAAGCGAATCAACGGTGAGTGGGTCTCAACGGATTTTAAGGATATGCCAGTCAACGAGAACACTGGTGAGTTCAAGCATAGCGATATGGTTAACTACGCCAATAAGAAGCTCGCTGAGATTGACAGTATGGACATTCCAGACGGTGCCAAGGATGCTATGAAGTTGAAGTACCTTCAAGCGGACTCTAAGGACGGAGAATTCCGTAc                                                                                                                                                                                                                                                                                             <  1:162733/301‑1 (MQ=255)`  
                  `ggATGAACGCATGGACGAAAGCTCAGGCCAAGGCTCTGGACGATTCCATGAAGTCAATGAACAAACTTGACGTAATCGACAAGCAATTCCAGAAGCGAATCAACGGTGAGTGGGTCTCAACGGATTTTAAGGATATGCCAGTCAACGAGAACACTGGTGAGTTCAAGTATAGCGATATGGTTAACTACGCCAATAAGAAGCTCGCTGAGATTGACAGTATGGACATTCCAGACGGTGCCAAGGATGCTATGAAGTTGAAGTACCTTCAAGCGGACTCTAAGGGCGGAGCATTCCGTACAGc                                                                                                                                                                                                                                                                                          <  1:95323/300‑1 (MQ=255)`  
                   `gATGAACGCATGGACGAAAGCTCAGGCCAAGGCTCTGGACGATTCCATGAAGTAAATGAACAAACTTGACGTAATCGACAAGCAATTCCAGAAGCGAATCAACGGTGAGTGGGTCTCAACGGATTTTAAGGATATGCCAGTCAACGAGAACACTGGTGAGTTCAAGCATAGCGATATGGTTAACTACGCCAATAAGAAGCTCGCTGAGATTGACAGTATGGACATTCCAGACGGTGCCAAGGATGCTATGAAGTTGAAGTACCTTCAAGCGGACTCTAAGGActgtctcttatacacatct                                                                                                                                                                                                                                                                                         >  1:218192/1‑283 (MQ=255)`  
                                 `agAAAGCTCAGGCCAAGGCTCTGGACGATTCCATGAAGTCAATGAACAAACTTGACGTAATCGACAAGCAATTCCAGAAGCGAATCAACGGTGAGTGGGTCTCAACGGATTTTAAGGATATGCCAGTCAACGAGAACACTGGTGAGTTCAAGCATAGCGATATGGTTAACTACGCCAATAAGAAGCTCGCTGAGATTGACAGTATGGACATTCCAGACGGTGCCAAGGATGCTATGAAGTTGAAGTACCTTCAAGCGGACTCTAAGGGCGGAGCATTCCGTACAGCCATCGGAACCATGGt                                                                                                                                                                                                                                                                           <  1:286744/300‑1 (MQ=255)`  
                                      `gCTCAGGCCAAGGCTCTGGACGATTCCATGAAGTCAATGAACAAACTTGACGTAATCGACAAGCAATTCCAGAAGCGAATCAACGGTGAGTGGGTCTCAACGGATTTTAAGGATATGCCAGTCAACGAGAACACTGGTGAGTTCAAGCATAGCGATATGGTTAACTACGCCAATACGAAGCTCGCTGAGATTGACAGTATGGACATTCCAGACGGTGCCAAGGATGCTATGAAGTTGAAGTACCTTCAATCGGACTCTAAGGACGGAGAATTCCGTACAGCCATCGGAACCATGGTCACTg                                                                                                                                                                                                                                                                      >  1:92524/1‑301 (MQ=255)`  
                                          `aGGCCAAGGCTCTGGACGATTCCATGAAGTCAATGAACAAACTTGACGAAATCGACAAGCAATTCCAGAAGCGAATCAACGGTGAGTGGGTCTCAACGGATTTTAAGGATATGCCAGTCAACGAGAACACTGGTGAGTTCAAGCATAGCGATGTGGTTAACTACGCCAATAAGAAGCGCGCTGAGATTGACAGTATGGACATTCCAGACGGTGCCAAGGATGCTATGAAGTTGAAGTACCTTCAAGCGGACTCTAAGGACGGAGCATTCAGTACAGCCATCGGAACCATGGTCACTGACGc                                                                                                                                                                                                                                                                  <  1:170153/301‑1 (MQ=255)`  
                                                  `gCTCTGGACGATTCCATGAAGTCAATGAACAAACTTGACGTAATCGACAAGCAATTCCAGAAGCGAATCAACGGTGAGTGGGTCTCAACGGATTTTAAGGATATGCCAGTCAACGAGAACACTGGTGAGTTCAAGCATAGCGATATGGTTAACTACGCCAATAAGAAGCTCGCTGAGATTGACAGTATGGATATTCCAGACGGTGCCAAGGATGCTATGACGTTGAAGTACCTTCAAGCGGACTCTAAGGGCGGAGCATTCCGTACAGCCACCGGAACCATGGTCACTTACGCTGGTCAag                                                                                                                                                                                                                                                          >  1:66365/1‑301 (MQ=255)`  
                                                  `gCTCTGGACGATTCCATGAAGTCAACGAACAAACTTGACGTAATCGACAAGCAATTCCAGAAGCGAATCAACGGTGAGTGGGTCTCAACGTATTTTAAGTATATGCCAGTCAACGAGAACACTGGTGAGTTCATGCATAGCGATATGGTTAACTACCCCAATAAGAAGCTCGCTGAGATTGACAGTATGGACATTCCAGACGGTGCCAAGGATGCTATGAAGTTGACGTACCTTCAAGCTGACTCTAAGGTCGGAGCATTCCGTACAGCCATCGGTACCATGTTAACTGACGCTGGTCaat                                                                                                                                                                                                                                                          >  1:127397/1‑300 (MQ=255)`  
                                                    `aaagagacagtCCATGAAGTCAATGAACAAACTTGACGTAATCGACAAGCAATTCCAGAAGCGAATCAACGGTGAGTGGGTCTCAACGGATTTTAAGGATATGCCAGTCAACGAGAACACTGGTGAGTTCAAGCATAGCGATATGGTTAACTACGCCAATAAGAAGCTCGCTGAGATTGACAGTATGGACATTCCAGACGGTGCCAAGGATGCTATGAAGTTGAAGTACATTCAAGCGGACTCTAAGGACGGAGAATTCCGTACAGCCATCGGAACCATGGTCACTGACGCTGGTCAagag                                                                                                                                                                                                                                                        <  1:84451/291‑1 (MQ=255)`  
                                                       `ggACGATTCCATGAAGTCAATGAACAAACTTGACGTAATCGACAAGCAATTCCAGAAGCGAATCAACGGTGAGTGGGTCTCAACGGATTTTAAGGATATGCCAGTCAACGAGAACACTGGTGAGTTCAAGCATAGCGATATGGTTAACTACGCCAATAAGAAGCTCGCTGAGATTGACAGTATGGACATTCCAGACGGTGCCAAGGATGCTATGAAGTTGAAGTACCTTCAAGCGGACTCTAAGGGCGGAGCATTCCGTACAGCCATCGGAACCATGGTCACTGACGCTGGTCAAGCGTgg                                                                                                                                                                                                                                                     >  1:177976/1‑301 (MQ=255)`  
                                                          `agATTCCATGAAGTCAATGAACAAACTTGACGTAATCGACAAGCAATTCCAGAAGCGAATCAACGGTGAGTGGGTCTCAACGGATTTTAAGGATATGCCAGTCAACGAGAACACTGGTGAGTTCAAGCATAGCGATATGGTTAACTACGCCAATAAGAAGCTCGCTGAGATTGACAGTATAGACATTCCAGACGGTGCCAAGGATGCTATGAAGTTGAAGTACCTTCAAGCGGACTCTAAGGGCGGAGCATTCCGTACAGCCATCGGAACCATGGTCACTGACGCTGGTCAAGAGTGGTCt                                                                                                                                                                                                                                                  <  1:271484/300‑1 (MQ=255)`  
                                                            `aTTCCATGAAGTCAATGAACAAACTTGACGTAATCGACAAGCAATTCCAGAAGCGAATCAACGGTGAGTGGGTCTCAACGGATTTTAAGGATATGCCAGTCAACGAGAACACTGGTGAGTTCAAGCATAGCGATATGGTTAACTACGCCAATAAGAAGCTCGCTGAGATTGACAGTATGGACATTCCAGACGGTGCCAAGGATGCTATGAAGTTGAAGTACCTTCAAGCGGACTCTAAGGGCGGAGCATTCCGTACAGCCATCGGAACCATGGTCACTGACGCTGGTCAAGAGTGGTCTGc                                                                                                                                                                                                                                                >  1:241482/1‑301 (MQ=255)`  
                                                               `ccATGAAGTCAATGAACAAACTTGACGTAATCGACAAGCAATTCCAGAAGCGAATCAACGGTTAGTGGGTCTCAACGGATTTTAAGGATATGCCAGTCAACGAGAACACTGGTGAGTTCAAGCATAGCGATATGGTTAACTACGCCAATAAGAAGCTCGCTGAGATTGACAGTATGGACATTCCTGACGGTGCCAAGGATGCTATGAAGTTGAAGTACCTTCAAGCGGACTCTAAGGGCGGAGCATTCCGTACAGCCATCGGAACCATGGTCACTGACGCTGGTCAAGAGTGGTCTGCCGc                                                                                                                                                                                                                                             <  1:42368/301‑1 (MQ=255)`  
                                                                      `gTCAATGAACAAACTTGACGTAATCGACGAGCAATTCCAGAAGCGAATCAACGGTGAGTGGGTCTCAACGGATTTTAAGGATATGCCAGTCAACGAGAACACTGGTGAGTTCAAGCATAGCGATATGGTTAACTACGCCAATAAGAAGCTCGCTGAGATTGACAGTATGGACATTCCAGACGGTGCCAAGGATGCTATGAAGTTGAAGTACCTTCAAGCGGACTCTAAGGACGGAGCATTCCGTACAGCCATCGGAACCATGGTCACTGACGCTGGTCAAGAGTGGTCTGCCGCTGTGAtt                                                                                                                                                                                                                                      <  1:103712/301‑1 (MQ=255)`  
                                                                            `gAACAAACTTGACGTAATCGACAAGCAATTCCAGAAGCGAATCAACGGTGAGTGGGTCTCAACGGATTTTAAGGATATGCCAGTCAACGAGAACACTGGTGAGTTCAAGCATAGCGATATGGTTAACTACGCCAATAAGAAGCTCGCTGAGATTGACAGTATGGACATTCCAGACGGTGCCAAGGATGCTATGAAGTTGAAGTACCTTCAAGCGGACTCTAAGGACGGAGAATTCCGTACAGCCATCGGAACCATGGTCACTGACGCTGGTCAAGAGTGGTCTGCCGCTGTGATTAACggc                                                                                                                                                                                                                                >  1:22441/1‑300 (MQ=255)`  
                                                                                `aaaCGTGACGTAAACGACAAACAATTCCAGAAGCGAATCAACGGTGAGTGGGTCTCAACGGATTTTAAGGATATGCCAATCAACTAGAACACTGGTGAGTTCAAGCATAGCGATATGGTTAACTAAGCCAATAAGAAGCTCGCTGAGATTGACAGTATGGACATTCCAGACGGTGCCAAGGATGCTATGAAGTTGAAGTACCTTCAAGCGGACTCTAAGGGCGGAGCATTCCGTACAGCCATCGGAACCATGGTCACTGACGCTGGTCAAGAGTGGTCTGCCGCTGTGATTACCGGTAAGt                                                                                                                                                                                                                            <  1:81666/301‑1 (MQ=255)`  
                                                                                 `aaCTTGACGTAATCGACAAGCAATTCCAGAAGCGAATCAACGGTGAGTGGGTCTCAACGGATTTTAAGGATATGCCAGTCAACGAGAACACTGGTGAGTTCAAGCATAGCGATATGGTTAACTACGCCAATAAGAAGCTCGCTTAGATTGACAGTATGGACATTCCAGACGGTGCCAAGGATGCTATGAAGTTGAAGTACCTTCAAGCGGACTCTAAGGACGGAGAATTCCGTACAGCCATCGGAACCATGGTCACTGACGCTGGTCAAGAGTGGTCTGCCGCTGTGATTAACGGTAAGtt                                                                                                                                                                                                                           >  1:268348/1‑301 (MQ=255)`  
                                                                                      `gACGTAATCGACAAGCAATTCCAGAAGCGAATCAACGGTGAGTGGGTCTCAACGGATTTTAAGGATATGCCAGTCAACGAGAACACTGGTGAGTTCAAGCATAGCGATATTGTTAACTACGCCAATAAGAAGCTCGCTGAGATTGACAGTATGGACATTCCAGACGGTGCCAAGGATGCTATGAAGTTGAAGTACCTTCAAGCGGACTCTAAGGACGGAGAATTCCGTACAGCCATCGGAACCATGGTCACTGACGCTGGTCAAGAGTGGTCTGCCGCTGTGATTAACGGTAAGTTACCaa                                                                                                                                                                                                                      >  1:167479/1‑300 (MQ=255)`  
                                                                                       `aCGTAATCGACAAGCAATTCCAGAAGCGAATCAACGGTGAGTGGGTCTCAACGGATTTTAAGGATATGCCAGTCAACGAGAACACTGGTGAGTTCAAGCATAGCGATATGGTTAACTACGCCAATAAGAAGCTCGCTGAGATTGACAGTATGGACATTCCAGACGGTGCCAAGGATGCTATGAAGTTGAAGTACCTTCAAGCGGACTCTAAGGGCGGAGCATTCCGTACAGCCATCGGAACCATGGTCACTGACGCTGGTCAAGAGTGGGCTGCCGCTGTGATTAACGGTAAGTTACCAga                                                                                                                                                                                                                     >  1:206992/1‑301 (MQ=255)`  
                                                                                                `aCAAGCAATTCCAGAAGCGAATCAACGGTGAGTGGGTCTCAACGGATTTTAAGGATATGCCAGTCAACGAGAACACTGGTGAGTTCAAGCATAGCGATATGGTTAACTACGCCAATAAGAAGCTCGCTGAGATTGACAGTATGGACATTCCAGACGGTGCCAAGGATGCTATGAAGTTGAAGTACCTTCAAGCGGACTCTAAGGACGGAGCATTCCGTACAGCCATCGGAACCATGGTCACTGACGCTGGTCAAGAGTGGTCTGCCGCTGTGATTAACGGTAAGTTACCCGAACGAAcccc                                                                                                                                                                                                            >  1:254371/1‑301 (MQ=255)`  
                                                                                                     `aagttCCAGAAGCGAATCAACGGTGAGTGGGTCTCAACGGATTTTAAGGATATGCCAGTCAACGAGAACACTGGTGAGTTCAAGCATAGCGATATGGTTAACTACGCCAATGAGAAGCTCGCTGAGATTGACAGTATGGACATTCCAGACGGTGCCAAGGATGCTATGAAGTTGAAGTACCTTCAAGCGGACTCTAAGGACGGAGCATTCCGTACAGCCATCGGAACCATGGTCACTGACGCTGGTCAAGAGTGGTCTGCCGCTGTGATTAACGGTAAGTTACCAGAACGAACCCCAGCTa                                                                                                                                                                                                       <  1:315637/298‑1 (MQ=255)`  
                                                                                                          `ccAGAAGCGAATCAACGGTGAGTGGGTCTCAACGGATTTTAAGGATATGCCAGTCAACGAGAACACTGGTGAGTTCAAGCATAGCGATATGGTTAACTACGCCAATAAGAAGCTCGCTGAGATTGACAGTATGGACATTCCAGACGGTGCCAAGGATGCTATGAAGTTGAAGTACCTTCAAGCGGACTCTAAGGACGGAGTATTCCGTACAGCCATCGGAACCATGGTCACTGACGCTGGTCAAGAGTGGTCTGCCGCTGTGATTAACGGTAAGTTACCAGAACGAACCCCAGCTATGGAt                                                                                                                                                                                                  <  1:157412/301‑1 (MQ=255)`  
                                                                                                          `ccAGAAGCGAATCAACGGTGAGTGGGTCTCAACGGATTTTAAGGATAAGCCAGTCAACGAGAACACTGGTGAGTTCAAGCATAGCGATATGGTTAACTACGCCAATAAGAAGCTCGCTGAGATTGACAGTATGGACATTCCAGACGGTGCCAAGGATGCTATGAAGTTGAAGTACCTACAAGCGGACTCTAAGGGCGGAGCATTCCGTACAGCCATCGGAACCATGGTCACTGACGCTGGTCAAGAGTGGTCTGCCGCTGTGATTAACGGTAAGTTACCAGAACGAACCCCAGCTATGGAt                                                                                                                                                                                                  <  1:69654/301‑1 (MQ=255)`  
                                                                                                          `aagagacagAATAAACGGTGAGTGGGTCTCAACGTATTTTAAGGATATGCCAGTCAACGAGAACACGGGTGAGTTCAAGCATAGCGATATGGTTAACTACGCCAATAAGAAGCTCGCTGAGATTGACAGTATGGACATTCCAGACGGTGCCAAGGATGCTATGAAGTTGAAGTACCTTCAAGCGGACTCTAAGTACGGAGAATTCCGTACAGCCATCGGAACCATGGTCACTGACGCTGGTCAAGAGTGGTCTGCCGCTGTGATTAACGGTAAGTTACCAGAACGAACCCCAGCTATGGAt                                                                                                                                                                                                  <  1:196263/293‑1 (MQ=255)`  
                                                                                                                   `aagcAACGGTGAGTGTGTCTCAACTGATTTTAAGGATATGCCAGTCAACGAGAACACTGGTGAGTTCAAGCATATCGAGATGGTTAACTACGCCAATAAGAAGCTCGCTGAGATTGACAGTATGGACATTCCAGACGGTGCCAAGGATGCTATGAAGTTGAAGTACCTTCAAGCGGACTCTAAGGACGGAGCATTCAGTACAGCCATCGGAACCATGGTCACTGACGCTGGTCAAGAGTGGTCTTCCGCTGTGATTAACGGTAAGTTACCAGAACGAACCCCAGCTATGGATGCTCTgcgc                                                                                                                                                                                         <  1:328501/298‑1 (MQ=255)`  
                                                                                                                   `aaTCAACGGTGAGTGGGTCTCAACGGATTTGAAGGATATGCCAGTCAACGAGAACACTGGTGAGTTCAAGCATAGCGATATGGTTAACTACGCCAATAAGAAGCTCGCTGAGATTGACAGTATGGACATTCCAGACGGTGCCAATGATGCTATGAAGTTGAAGTACCTTCAAGCGGACTCTAAGGACGGAGCATTCCGTACAGCCATCGGAACCATGGTCACTGACGCTGGTCAAGAGTGGTCTGCCGCTGTGATTAACGGTAAGTTACCAGAACGAACCCCAGCTATGGATGCTATgcgc                                                                                                                                                                                         <  1:125217/301‑1 (MQ=255)`  
                                                                                                                       `aaCGGTGAGTGGGTCTCAACGGATTTTAAGGATATGCCAGTCAACGAGAACACTGGTGAGTTCAAGCATAGCGATATGGTTAACTACGCCAATAAGAAGCTCGCTGAGATTGACAGTAGGGACATTCCAGACGGTGCCAAGGATGCTATGAAGTTGAAGTACCTTCAAGTGGACTCTAAGGACGGAGCATTCCGTGCAGCCATCGGAACCATGGTCACTGACGCTGGTCAAGAGTGGTCTGCCGCTGTGATTAACGGTAAGTTACCAGAACGAACCCCAGCTATGGATGCTCTGCGCAGaa                                                                                                                                                                                     <  1:249056/301‑1 (MQ=255)`  
                                                                                                                        `aCGGTGAGTGGGTCTCAACGGATTTTAAGGATATGCCAGTCAACGAGAACACTGGTGAGTTCAAGCATAGCGATATGGTTAACTACGCCAATAAGAAGCTCGCTGAGATTGACAGTATGGACATTCCAGACGGTGCCAAGGATGCTATGAAGTTGAAGTACCTTCAAGCGGACTCTAAGGGCGGAGCATTCCGTACAGCCATCGGAACCATGGTCACTGACGCTGGTCAAGAGTGGTCTGCCGCTGTGATTAACGGTAAGTTACCAGAACGAACCCAAGCTATGGATGCTCTGCGCAGAAt                                                                                                                                                                                    <  1:22066/301‑1 (MQ=255)`  
                                                                                                                          `ggTGAGTGGGTCTCAACGGATTTTAAGGATATGCCAGTCAACGAGAACACTGGTGAGTTCAAGCATGGCGATATGGTTAACTACGCCAATAAGAAGCTCGCTGAGATTGACAGTATGGACATTCCAGACGGTGCCAAGGATGCTATGAAGTTGAAGTACCTTCAAGCGGACTCTAAGGGCGGAGCATTCCGTACAGCCATCGGAACCATGGTCACTGACGCTGGTCAAGAGTGGTCTGCCGCTGTGATTAACGGTAAGTTACCAGAACGAACCCCAGCTATGGATGCTCTGCGCAGAATcc                                                                                                                                                                                  >  1:164666/1‑301 (MQ=255)`  
                                                                                                                          `ggTGAGTGGGTCTCAACGGATTTTAAGGATATGCCAGTCAACGAGAACACTGGTGAGTTCAAGCATAGCGATATGGTTAACTACGCCAATAAGAAGCTCGCTGAGATTGACAGTATGGACATTCCAGACGGTGCCAAGGATGCTATGAAGTTGAAGTACCTTCAAGCGGACTCTAAGGGCGGAGCATTCCGTACAGCCATCGGAACCATGGTCACTGACGCTGGTCAAGAGTGGGCTGCCGCTGTGATTAACGGTAAGTTACCAGAACGAACCCCAGCTATGGATGCTCTGCGCAGAATcc                                                                                                                                                                                  >  1:303102/1‑301 (MQ=255)`  
                                                                                                                           `gTGCGTGGGTCTCAACGGATTTTAAGGATATGCCAGTCAACGCTCACCCTGTTGAGCTCAAGCATATCGATATGGTTAACTACGCCAATAAGAAGCTCGCTGAGATTGACAGTATGGACATTCCATACTGTGCCAATGATGCTATGAAGCTGTAGTACCTTCCAGCGGCCACTAAGGGCGGAGCATTCCCTACAGCCATCGGAACCATGGTCACTGACGCCGGTCAAGTGTGGCCCTCCGCCGTGATTAACGGTATGTTACCAGAACGAACCCCCGCTATAGATGCTCTGCGCAGAATTCg                                                                                                                                                                                 >  1:318333/1‑301 (MQ=255)`  
                                                                                                                           `gTGAGTGGGTCTCAACGGATTTTAAGGATATGCCAGTCAACGAGAACACTGGTGAGTTCAAGCATAGCGATATGGTTAACTACGCCAATAAGAAGCTCGCTGAGATTGACAGTATGGACATTCCAGACGGTGCCAAGGATGCTATGAAGTTGAAGTACCTTCAAGCGGACTCTAAGGACGGAGAATTCCGTACAGCCATCGGAACCATGGTCACTGACGCTGGTCAAGAGTGGTCTGCCGCTGTGATTAACGGTCAGTTACCAGAACGAACCCCAGCTATGGATGCTCTGCGCAGAATccc                                                                                                                                                                                 >  1:333267/1‑300 (MQ=255)`  
                                                                                                                             `gAGTGGGTCTCAACGGATTTTAAGGATATGCCAGTCAACGAGAACACTGGTAAGTTCAAGCATAGCGATATGGTTAACTACGCCAATAAGAAGCTCGCTGAGATTGACAGTATGGACATTCCAGACGGTGCCAAGGATGCTATGAAGTTGAAGTACCTTCAAGCGGACTCTAAGGACGGAGCATTCCGTACAGCCATCGGAACCATGGTCACTGACGCTGGTCAAGAGTGGTCTGCCGCTGTGATTAACGGTAAGTTACCAGAACGAACCCCAGCTactgtctcttatacacatctccgag                                                                                                                                                                               >  1:325084/1‑277 (MQ=255)`  
                                                                                                                               `gTGGGTCTCAACGGATTTTAAGGATATGCCAGTCAACGAGAACACTGGTGAGTTCAAGCATGGCGATATGGTTAACTACGCCAATAAGAAGCTCGCTGAGATTGACAGTATGGACATTCCAGACGGTGCCAAGGATGCTATGAAGTTGAAGTACCTTCAAGCGGACTCTAAGGGCGGAGCATTCCGTACAGCCATCGGAACCATGGTCACTGACGCTGGTCAAGAGTGGTCTGCCGCTGTGATTAACGGTAAGTTACCAGAACGAACCCCAGCTATGGATGCTCTGCGCAGAATCCGCAAt                                                                                                                                                                             <  1:133/301‑1 (MQ=255)`  
                                                                                                                                  `ggTCTCAACGGATTTTAAGGATATGCCAGTCAACGAGAACACTGGTGAGTTCAAGCATAGCGATATGGTTAACTACGCCAATAAGAAGCTCGCTGAGATTGACAGTATGGACATTCCAGACGGTGCCAAGGATGCTATGAAGTTGAAGTACCTTCAAGCGGACTCTAAGGACGGAGCATTCCGTACAGCCATCGGAACCATGGTCACTGACGCTGGTCAAGAGTGGTCTGCCGCTGTGATTAACGGTAAGTTACCAGAACGAACCCCAGCTATGGATGCTCTGCGCAGAATCCGCAATGCt                                                                                                                                                                          >  1:101613/1‑301 (MQ=255)`  
                                                                                                                                    `tctcAACGGATTTTAAGGATATGCCAGTCAACGAGAACACTGGTGAGTTCAAGCATAGCGATATGGTTAACTACGCCAATAAGAAGCTCGCTGAGATTGACAGTATGGACATTCCAGACGGTGCCAAGGATGCTATGAAGTTGAAGTACCTTCAAGCGGACTCTAAGGGCGGAGCATTCCGTACAGCCATCGGAACCATGGTCACTGACGCTGGTCAAGAGTGGTCTGCCGCTGTGATTAAAGGTAAGTTACCAGAACGAACCCCAGCTATGGATGCTCTGCGCAGAATCCGCAATGCTgc                                                                                                                                                                        >  1:181252/1‑300 (MQ=255)`  
                                                                                                                                            `gATTTTAAGGATATGCCAGTCAACGAGAACACTGGAGAGTTCAAGCATAGCGATATGGTTAACTACGCCAATAAGAAGCTCGCTGAGATTGACAGTATGGACATTTCAGACGGTGCCAAGGATGCTATGAAGTTGAAATACCTTCAAGCGGACTCTAAGGACGGAGAATTCCGTACAGCCATCGAAACCATGGTCACTGACGCTGGCCAAGAGTGGTCTGCCGCTGTGATTAACGGTAAGTCACCAGAACGAACTCCAGCTAGGGATGCTCTGCGCAGAATCCGCACTgtctcttctacac                                                                                                                                                                >  1:141544/1‑289 (MQ=255)`  
                                                                                                                                                   `aGGATACTCCAGTCAACGAGATCACTGGTGAGTTCAAGCATAGCGATATGGTTAACTACGCCAATAAGAAGCTCGCTGAGATTGACAGTATGGACATTCCAGACGGTGCCAAGGATGCTATGAAGTTGAAGTACCTTCAAGTGGACTCTAAGGGCGGAGCATTCCGTACAGCCATCGGAACCATGGTCACTGACGCTGGTCAAGAGTGGTCTGCCGCTGTGATTAACGGTAAGTTACCAGAACGAACCCCAGCTATGGATGCTCTGCGCAGAATCCGCAATGCTGACCCTCAGTTGATtgc                                                                                                                                                         <  1:289577/301‑1 (MQ=255)`  
                                                                                                                                                    `ggATATGCCAGTCAACGAGAACACTGGTGAGTTCAAGCATAGCGATATGGTTAACTACGCCAATAAGAAGCTCGCTGAGATTGACAGTATGGACATTCCAGACGGTGCCAAGGATGCTATGAAGTTGAAGTACTTTCAAGCGGACTCTAAGGACGGAGCATTCCGTACAGCAATCGGAACCATGGTCCCTGACGCTGGTTAAGAGTGGTCTGCCGCTGTGATTAACGGTAAGTTACCAGAACGAACCCCAGCTATGGATGCTCTGCGCAGAATCCGCAATGCTGACCCTCAGCTGATtgct                                                                                                                                                        >  1:260209/1‑301 (MQ=255)`  
                                                                                                                                                              `gTCAACGAGAACACTGGTGAGTTCAAGCATAGCGATATGGTTAACTATGCCAATAAGAAGCTCGCTGAGATTGACAGTATGGACATTCCAGACGGTGCCAAGGATGCTATGAAGTTGAAGTACCTTCAAGCGGACTCTAAGGGCGGAGTATTCCGTACAGCCATCGGAACCATGGTCACTGACGCTGGTCAAGAGTGGTCTGCCGCTGTGATTAACGGTAAGTTACCAGAACGAACCCCAGCTATGGATGCTCTGCGCAGAATCCGCAATGCTGACCCTCAGTTGATTGCTGCGCTATAcc                                                                                                                                              >  1:305508/1‑301 (MQ=255)`  
                                                                                                                                                              `gTCAACGAGAACACTGGTGAGTTCAAGCATAGCGATATGGTTAACTACGCCAATAAGAAGCTCGCTGAGATTGACAGTATGGACATTCCAGACGGTGCCAAGGATGCTATGAAGTTGAAGTACCTTCAAGCGGACTCTAAGTGCGGAGCATTCCGTACAGCCATCGGAACCATGGTCACTGACGCTGGTCAAGAGTGGTCTGCCGCTGTGATTAACGGTAAGTTACCAGAACGAACCCCAGCTATGGATGCTCTGCGCAGAATCCTCAATGCTGACCCTCAGTTGAttctgtctcttatac                                                                                                                                              >  1:321317/1‑288 (MQ=255)`  
                                                                                                                                                                           `cTGGTGAGTTCAAGCATAGCGATATGGTTAACTACGCCAATAAGAAGCTCGCTGAGATTGACAGTATGGACATTCCAGACGGTGCCAAGGATGCTATGAAGTTGAAGTACCTTCAAGCGGACTCTAAGGACGGAACATTCCGTACAGCCATCGGAACCATGGTCACTGACGCTGGTCAAGAGTGGTCTGCCGCTGTGATTAACGGTAAGTTACCAGAACGAACCCCAGATATGGATGCTCTGCGCAGAATCCGCAATGCTGACCCTCAGTTGATTGCTGCGCTATACCCAGACCAAGCTGa                                                                                                                                 >  1:282959/1‑301 (MQ=255)`  
                                                                                                                                                                             `ggTGAGTTCAAGCATAGCGATATGGTTAACTACGCCAATAAGAAGCTCGCTGAGATTGACAGTATGGACATTCCAGACGGTGCCAAGGATGCTATGAAGTTGAAGTACCTTCAAGCGGACTCTAAGGGCGGAGCATTCCGTACAGCCATCGGAACCATGGTCACTGACGCTGGTCAAGAGTGGTCTGCCGCTGTGATTAACGGTAAGTTACCAGAACGAACCCCAGCTATGGATGCTCTGCGCAGAATCCGCAATGCTGACCCTCAGTTGATTGCTGCGCTATAcccctgtctcttataca                                                                                                                               >  1:62402/1‑287 (MQ=255)`  
                                                                                                                                                                               `agAGTGCAAGCATAGCGATATGGTTAACGACGCCAATAAGAAGCGCGCTGAGATTGACAGTATGGACATTCCAGACGGTGCCAAGGATGCTATGAAGTTGAAGTACCTTCAAGCGGACTCTAAGGACGGAGAATTCCGTACAGCCATCGGAGCCATGGTCACTGACGCTGGTCAAGAGTGGTCTGCCGCTGTGATTAACGGTAAGTTACCAGAACGAACCCCAGCTATGGATGCTCTGCGCAGAATCCGCAATGCTGACCCTCAGTTGATTGCTGCGCTATACCCAGACCAAGCTGAGCTa                                                                                                                             <  1:75645/300‑1 (MQ=255)`  
                                                                                                                                                                                  `gTTCAAGCATAGCGATATGGTTAACTACGCCAATAAGAAGCTCGCTGAGATTGACAGTATGGACATTCCAGACGGTGCCAAGGATGCTATGAAGTTGAAGTACCTTCAAGCGGACTCTAAGGGCGGAGCATTCCATACAGCCATCGGAACCATGGTCACTGACGCTGGTCAAGAGTGGTCTGCCGCTGTGATTAACGGTAAGTTACCAGAACGAACCCCAGCTATGGATGCTCTGCGCAGAATCCGCAATGCTGACCCTCAGTTGATTGCTGCGCTATACCCAGACCAAGCTGAGCTATTc                                                                                                                          >  1:121909/1‑301 (MQ=255)`  
                                                                                                                                                                                  `gTTCAAGCATAGCGATATGGTTAACTACGCCAATAAGAAGCTCGCTGAGATTGACAGTATGGACATTCCAGACGGTGCCAAGGATGCTATGAAGTTGAAGTACCTTCAAGCGGACTCTAAGGGCGGAGCATACCGTACAGCCATCGGAACCATGGTCACTGACGCTGGTCAAGAGTGGTCTGACGCTGTGATTAACGGTAAGTTACCAGAACGAACCCCAGCTATGGATGCTCTGCGCAGAATCCGCAATGCTGACCCTCAGTTGATTGCTGCGCTATACCCAGACCAAGCTGAGCTATTc                                                                                                                          <  1:274124/301‑1 (MQ=255)`  
                                                                                                                                                                                        `gCATAGCGATATGGTTAACTACGCCAATAAGAAGCTCGCTGAGATTGACAGTATGGACATTCCAGACGGTGCCAAGGATGCTATGAAGTTGAAGTACCTTCAAGCGGACTCTAAGGGCGGAGCATTCCGTACAGCCATCGGAACCATGGTCACTGACGCTGGTCAAGAGTGGTCTGCCGCTGTGATTAACGGTAAGTTACCAGAACGAACCCCAGCTATGGATGCTCTGCGCAGAATCCGCAATGCTGACCCTCAGTTGATTGCTGCGCTATACCCAGACCAAGCTGAGCTATTCCTGACg                                                                                                                    <  1:311871/301‑1 (MQ=255)`  
                                                                                                                                                                                           `tAGCGATATTGTTAACTACGCCAATAAGAAGCTCGCTGAGATTGACAGTATGGACATTCCAGACGGTGCCAAGGATGCTATGAAGTTGAAGTACCTTCAAGCGGACTCTAAGGGCGGAGCATTCCGTACAGCCATCGGAACCATGGTCACTGACGCTGGTCAAGAGTGGTCTGCCGCTGTGATTAACGGTAAGTTACCAGAACGAACCCCAGCTATGGATGCTCTGCGCAGAATCCGCAATGCTGACCCTCAGTTGATTGCTGCGCTATACCCAGACCAAGCTGAGCTATTCCTGAcctgt                                                                                                                 >  1:175063/1‑297 (MQ=255)`  
                                                                                                                                                                                           `tAGCGATATGGTTAACTACGCCAATAAGAAGCTCGCTGAGATTGACAGTATGGACATTCCAGACGGTGCCAAGGATGCTATGAAGTTGAAGTACCTTCAAGCGGACTCTAAGGGCGGAGCATTCCGTACAGCCATCGGAACCATGGTCACTGACGCTGGTCAAGAGTGGTCTGCCGCTGTGATTAACGGTAAGTTACCAGAACGAACCCCAGCTATGCATGCTCTGCGCAGAATCCGCAATGCTGACCCTCAGTTGATTGCTGCGCTATACCCAGACCAAGCTGAGCTATTCCTGACGATg                                                                                                                 >  1:244276/1‑301 (MQ=255)`  
                                                                                                                                                                                             `gCGATATGGTTAACTACGCCAATAAGAATCTCGCTGAGATTGACAGTATGGACATTCCAGACGGTGCCAAGGATGCTATGAAGTTGAAGTACCTTCAAGCGGACTCTAAGGACGGAGAATTCCGTACAGCCATCGGAACCATGGTCACTGACGCTGGTCAAGAGTGGTCTGCCGCTGTGATTAACGGTAAGTTACCAGAACGAACCCCAGCTATGGATGCTCTGCGCAGAATCCGCAATGCTGACCCTCAGATGATTGCTGCGCTATACCCAGACCAAGCTGAGCTATTCCTGACGATggg                                                                                                               >  1:307155/1‑300 (MQ=255)`  
                                                                                                                                                                                               `gATATGGTTAACTACGCCAATAAGAAGCTCGCTGAGATTGACAGTATGGACATTCCAGACGGTGCCAAGGATGCTATGAAGTTGAAGTACCTTCAAGCGGACTCTAAGGGCGGAGCATTCCGTACAGCCATCGGAACCATGGTCACTGACGCTGGTCAAGAGTGGTCTGCCGCTGTGATTAACGGTAAGTTACCAGAACGAACCCCAGCTATGGATGCTCTGCGCAGAATCCGCAATGCTGACCCTCAGTTGATTGCTGCGCTATACCCAGACCAAGCTGAGCTATTCCTGACGATGGACa                                                                                                             <  1:53355/301‑1 (MQ=255)`  
                                                                                                                                                                                               `aagatGGTTAACTACGCCAATAAGAAGCTCGCTGAGATTGACAGTATGGACATTCCAGACGGTGCCAAGGATGCTATGAAGTTGAAGTACCTTCAAGCGGACTCTAAGGACGGAGAATTCCGTACAGCCATCGGAACCATGGTCACTGACGCTGGTCAAGAGTGGTCTGCCGCTGTGATTAACGGTAAGTTACCAGAACGAACCACAGCTATGGATGCTCTGCGCAGAATCCGCAATGCTGACCCTCAGTTGATTGCTGCGCTATACCCAGACCAAGCTGAGCTATTCCTGACGATGGACa                                                                                                             <  1:213097/298‑1 (MQ=255)`  
                                                                                                                                                                                                  `atGGTTAACTACGCCAATAAGAAGCTCGCTGAGATTGACAGTATGGACATTCCAGACGGTGCCAAGGATGCTATGAAGTTGAAGTACCTTCAAGCGGACTCTAAGGGCGGAGCATTCCGTACAGCCATCGGAACCATGGTCACTGACGCTGGTCAAGAGTGGTCTGCCGCTGTGATTAACGGTAAGTTACCAGAACGAACCCCAGCTATGGATGCTCTGCGCAGAATCCGCAATGCTGACCCTCAGTTGATTGCTGCGCTATACCCAGACCAAGCTGAGCTATTCCTGACGATGGACatga                                                                                                          >  1:215112/1‑301 (MQ=255)`  
                                                                                                                                                                                                    `ggTTAACTACGCCAATAAGAAGCTCGCTGAGATTGACAGTATGGACATTCCAGACGGTGCCAAGGATGCTATGAAGTTGAAGTACCTTCAAGCGGACTCTAAGGGCGGAGCATTCCGTACAGCCATCGGAACCATGGTCACTGACGCTGGTCAAGAGTGGTCTGCCGCTGTGATTAACGGTAAGTTACCAGAACGAACCCCAGCTATGGATGCTCTGCGCAGAATCCGCAATGCTGACCCTCAGTTGATTGCTGCGCTATACCCAGACCAAGCTGAGCTATTCCTGACGATGGACatgatg                                                                                                        >  1:183699/1‑301 (MQ=255)`  
                                                                                                                                                                                                    `ggTTAACTACGCCAATAAGAAGCTCGCTGAGATTGACAGTATGGACATTCCAGACGGTGCCAAGGATGCTATGAAGTTGAAGTACCTTCAAGCGGACTCTAAGGACGGAGTATTCCGTACAGCCATCGGAACCATGGTCACTGACGCTGGTCAAGAGTGGTCTGCCGCTGTGATTAACGGTAAGTTACCAGAACGAACCCCAGCTATGGCTGCTCTGCGCAGAATCCGCAATGCTGACCCTCAGTTGATTGCTGCGCTATACCCAGACCAAGCTGAGCTATTCCTGAcctgtctcttatac                                                                                                        >  1:88289/1‑288 (MQ=255)`  
                                                                                                                                                                                                       `tAACTACGCCAATAAGAAGCTCGCTGAGATGGACAGTATGGACATTCCAGACGGTGCCAAGGATGCTATGAAGTTGAAGTACCTTCAAGCGGACTCTAATGACGGAGAATTCCGTACAGCCATCGGAACCATGGTCACTGACGCTGGTCAAGAGTGGTCTGCCGCTGTGATTAACGGTAAGTTACCAGAACGAACCCCAGCTATGGATGCTCTGCGCAGAATCCGCAATGCTGACCCTCAGTTGATTGCTGCGCTATACCCAGACCAAGCTGAGCTATTCCTGACGATGGACATGATGGAc                                                                                                     <  1:341530/301‑1 (MQ=255)`  
                                                                                                                                                                                                        `acaggcgacAATAAGAAGCTCGCTGAGATTGACAGTATGGACATTCCAGGCGGTGCCAAGGATGCTATGAACTTGAAGTACCGTCAAGCGGACTCTAAGGACGGAGCATTCCGTACAGCCATCGGAACCATGGTCACTGACGCTGGTCAAGAGTGGTCTGCCGCTGTGATTAACGGTAAGTTACCAGAACGAACCCCAGCTATGGATGCTCTGCGCAGAAGCCGCAATGCTGACCATCAGTTGATTGCTGCGCTATACCCAGACCAAGCTGAGCTATCCCTGACGATGGACATGATGGACa                                                                                                    <  1:298040/293‑1 (MQ=255)`  
                                                                                                                                                                                                        `aaCTACGCCAATAAGAAGCTCGCTGAGATTGACAGTATGGACATTCCAGACGGTGCCAAGGATGCTATGAAGTTGAAGTACCTTCAAGCGGACTCTAAGGGCGGAGCATTCCGTACAGCCATCGGAACCATGGTCACTGACGCTGGTCAAGAGTGGTCTGCCGCTGTTATTAACGGTAAGTTACCAGAACGAACCCCAGCTATGGATGCTCTGCGCAGAATCCGCAATGCTGACCCTCAGTTGATTGCTGCGCTATACCCAGACCAAGCTGAGCTATTCCTGACGATGGACATGATGGACa                                                                                                    >  1:80093/1‑301 (MQ=255)`  
                                                                                                                                                                                                         `aCTACGCCAATAAGAAGCTCGCTGATATTGACAGTATGGACATTCCAGACGGTGCCAAGGATGCTATGAAGTTGAAGTACCTTCAAGCTGACTCTAAGGGCGGAGCATTCCGTACAGCCATCGGAACCATGGTCACTGACGCTGGTCAAGAGTGGTCTGCCGCTGTGATTAACGGTAAGTTACCAGAACGAACCCCAGCTATGGATGCTCTGCGCAGAATCCGCAATGCTGACCCTCAGTTGATTGCTGCGCTATACCCAGACCAAGCTGAGCTATTCCTGACGATGGACATGATGGACaa                                                                                                   <  1:100004/301‑1 (MQ=255)`  
                                                                                                                                                                                                                  `aTAAGAAGCTCGCTGAGATTGACAGTATGGACATTCCAGACGGTGCCAAGGATGCTATGAAGTTGAAGTACCTTCAAGCGGACTCTAAGGGCGGAGCATTCCGTACAGCCATCGGAACCATGGTCACTGACGCTGGTCAAGAGTGGTCTGCCGCTGTGATTAACGGTAAGTTACCAGAACGAACCCCAGCTATGGATGCTCTGCGCAGAATCCGCAATGCTGACCCTCAGTTGATTGCTGCGCTATACCCAGACCAAGCTGAGCTATTCCTGACGATGGACATGATGGACAAGCAGGGTAt                                                                                          <  1:251823/301‑1 (MQ=255)`  
                                                                                                                                                                                                                  `aTAAGAAGCTCGCTGAGATTGACAGTATGGACATTCCAGACGGTGCCAAGGATGCTATGAAGTTGAAGTACCTTCAAGCGGACTCTAAGGACGGAGAATTCCGTACAGCCATCGGAACCATGGTCACTGACGCTGGTCAAGAGTGGTCTGCCGCTGTGATTAACGGTAAGTTACCAGAACGAACCCCAGCTATGGATGCTCTGCGCAGAATCCGCAATGCTGACCCTCAGTTGATTGCTGCGCTATACCCAGACCAAGCTGAGCTATTCCTGACGATGGACATGATGGACAAGCAGGGTAt                                                                                          <  1:19224/301‑1 (MQ=255)`  
                                                                                                                                                                                                                       `acgcacGCTGAGATTGACAGTATGTACATTCCAGACTGTGCCAAGGATGCGATGAAGTTGAAGTACCGTCAAGCGGACTCTAAGGACGGAGAATTCCGTACAGCCATCGGAACCATGTTAACTGACGCTGGTCAAGAGTGGTCTGCCGCTGTGATTAACGGTAAGTTACCAGAACGAACCCCAGCTATGGATGCTATGCGCAGAATCCGCAATGCTGACCCTCAGTTGATTGCTGCGCTATACCCAGACCAAGCTGAGCTATTTCTGACGATGTACATGATGGACAAGCAGGGTATTGAcc                                                                                     <  1:330423/296‑1 (MQ=255)`  
                                                                                                                                                                                                                       `aagCTCGCTGAGATTGACAGTATGGACATTCCAGACGGTGCCAAGGATGCTATGAAGTTGAAGTACCTTCAAGCGGACTCTAAGGGCGGAGCATTCCGTACAGCCATCGGAACCATGGTCACTGACGCTGGTCAAGAGTGGTCTGCCGCTGTGATTAACGGTAAGTTACCAGAACGAACCCCAGCTATGGATGCTCTGCGCAGAATCCGCAATGCTGACCCTCAGTTGATTGCTGCGCTATACCCAGACCAAGCTGAGCTATTCCTGACGATGGACATGATGGACAAGCAGGGTATTGAcc                                                                                     <  1:135077/301‑1 (MQ=255)`  
                                                                                                                                                                                                                         `gCTCGCTGAGATTTACAGTATGGACATTCCAGACGGTGCCAAGGATGCTATGAAGTTGAAGTACCTTCAAGCGGACTCTAAGGGCGGAGCATTCCGTACAGCCATCGGAACCATGGTCACTGACGCTGGTCAAGAGTGGTCCGCCGCTGTGATTAACGGTAAGTTACCAGAACGAACCCCAGCTATGGATGCTCTGCGCAGAATCCGCAATGCTGACCCTCAGTTGATTGCTGCGCTATACCCAGACCAAGCTGAGCTATTCCTGACGATGGACATGATGGACAAGCAGGGTATTGAcccc                                                                                   >  1:12962/1‑300 (MQ=255)`  
                                                                                                                                                                                                                            `cGCTGAGATTGACAGTATGGACATTCCAGACGGTGCCAAAGATGCTATGAAGTTGAAGTACCTTCAAGCGGACTCTAAGGGCGGAGCATTCCGTACAGCCATCGGAACCATGGTCACTGACGCTGGTCAAGAGTGGTCTGCCGCTGTGATTAACGGTAAGTTACCAGAACGAACCCCAGCTATGGATGCTCTGCGCAGAATCCGCAATGCTGACCCTCAGTTGATTGCTGCGCTATACCCAGACCAAGCTGAGCTATTCCTGACGATGGACATGATGGACAAGCAGGGTATTGACCCTCAg                                                                                >  1:112564/1‑301 (MQ=255)`  
                                                                                                                                                                                                                             `gtataagagacaggTATGGACATTCCAGACGGTGCCAAGGATGCTATGAAGTTGAAGTACCTTCAAGCGGACTCTAAGGACGGAGAATTCCGTACAGCCATCGGAACCATGGTCACTGACGCTGGTCAAGAGTGGTCTGCCGCTGTGATTAACGGTAAGTTACCAGAACGAACCCCAGCTATGGATGCTCTGCGCAGAATCCGCAATGCTGACCCTCAGTTGATTGCTGCGCTATACCCAGACCAAGCTGAGCTATTCCTGACGATGGACATGATGGACAAGCAGGGTATTGACCCTCAgg                                                                               <  1:58104/288‑1 (MQ=255)`  
                                                                                                                                                                                                                              `ggagtctggctaGTATGGACATTCCGGCCGGTGCCAAGGATGCTATGAAGTTGAAGTACCTTCAAGCGGACTCTAGGGACGGAGAATTCCGTACAGCCATCGGATCCATGGTCACTGACGCTGGTGAAGAGTGGTCTGCCGCTGTGATTAACGGTAAGTTACCAGAACGAACCCCAGCTATGGATGCTCTGCGCAGAATCCGCAATGCTGACCCTCAGTTGATTGCTGCGCTATACCCAGACCAAGCTGAGCTATTCCTGACGATGGGCATGATGGACAAGCAGGGTATTGACCCTCAGGt                                                                              <  1:109329/290‑1 (MQ=255)`  
                                                                                                                                                                                                                               `agagaTTGACAGTATGGACATTCCAGACGGTGCCAAGGATGCTATGAAGTTGAAGTACCTTCAAGCGGACTCTAAGGGCGGAGCATTCCGTACAGCCATCGGAACCATGGTCACTGACGCTGGTCAAGAGTGGTCTGCCGCTGTGATTAACGGTAAGTTACCAGAACGAACCCCAGCTATGGATGCTCTGCGCAGAATCCGCAATGCTGACCCTCAGTTGATTGATGCGCTATACCCAGACCAAGCTGAGCTATTCCTGACGATGGACATGATGGACAAGCAGGGTATTGACCCTCAGGtt                                                                             >  1:231572/2‑301 (MQ=255)`  
                                                                                                                                                                                                                                  `gaTTGACAGTATGGACATTCCAGACGGTGCCAAGGATGCTATGAAGTTGAAGTACCTCCAAGCGGACTCTAAGGGCGGAGCATTCCGTACAGCCATCGGAACCATGGTCACTGACGCTGGTCAAGAGTGGTCTGCCGCTGTGATTAACGGTAAGTTACCAGAACGAACCCCAGCTATGGATGCTCTGCGCAGAATCCGCAATGCTGACCCTCAGTTGATTGCTGCGCTATACCCAGACCAAGCTGAGCTATTCCTGACGATGGACATGATGGACAAGCAGGGTATTGACCCTCAGGTTAtt                                                                          >  1:47114/1‑301 (MQ=255)`  
                                                                                                                                                                                                                                    `tttaCAGTATGGACATTCCAGACGGTGCCAAGGATGCTATGAAGTTGAAGTACCTTCAAGCGGACTCTAAGGGCTGAGCATGCCGTACAGCCATCGGAACCATGGTCACTGACGCTGGTCAAGAGTGGTCTGCCGCTGTGATTAACGGTAAGTTAACAGAACGAACCCCAGCTATGGATGCTCTGCGCAGAATCCGCAATGCTGACCCTCAGTTGATTGCTGCGCTATACCCAGACCAAGCTGAGCTATTCCTGACGATGGACATGATGGACAAGCAGGGTATTGACCCTCAGGTTATTCt                                                                        <  1:50561/298‑1 (MQ=255)`  
                                                                                                                                                                                                                                    `ttGACAGTATGGACATTCCAGACGGTGCCAAGGATGCTATGAAGTTGAAGTACCTTCAAGCGGACTCTAAGGGCGGAGCATTCCGTACAGCCATCGGAACCATGGTCACTGACGCTGGTCAAGAGTGGTCTGCCGCTGTGATTAACGGTAAGTTACCAGAACGAACCCCAGCTATGGATGCTCTGCGCAGAATCCGCAATGCTGACCCTCAGTTGATTGCTGCGCTATACCCAGACCAAGCTGAGCTATTCCTGACGATGGACATGATGGACAAGCAGGGTAGTGACCCTCAGGTTATTCt                                                                        <  1:148394/301‑1 (MQ=255)`  
                                                                                                                                                                                                                                    `ttGACAGTATGGACATTCCAGACGGTGCCAAGGATGCTATGAAGTTGAAGTACCTTCAAGCGGACTCTAAGGGCGGAGCATTCCGTACAGCCATCGGAACCATGGTCACTGACGCTGGTCAAGAGTGGTCTGCCGCTGTGATTAACGGTAAGTTACCAGAACGAACCCCAGCTATGGATGCTCTGCGCAGAATCCGCAATGCTGACCCTCAGTTGATTGCTGCGCTATACACAGACCAAGCTGAGCTATTCCTGACGATGGACATGATGGACAAGCAGGGTATTGACCCTCAGGTTATTCt                                                                        <  1:30992/301‑1 (MQ=255)`  
                                                                                                                                                                                                                                    `cagACAGTATGGACATTCCAGACGGTGCCAAGGATGCTATGAAGTTGAAGTACCTTCAAGCGGACTCTAAGGGCGGAGCATTCCGTACAGCCATCGGAACCATGGTCACTGACGCTGGTCAAGAGTGGTCTGCCGCTGTGATTAACGGTAAGTTACCAGAACGAACCCCAGCTATGGATGCTCTGCGCAGAATCCGCAATGCTGACCCTCAGTTGATTGCTGCGCTATACCCAGACCAAGCTGAGCTATTCCTGACGATGGACATGATGGACAAGCAGGGTATTGACCCTCAGGTTATTCt                                                                        <  1:209896/299‑1 (MQ=255)`  
                                                                                                                                                                                                                                       `aCAGTATGGACATTCCAGACGGTGCCAAGGATGCTATGAAGTTGAAGTACCTTCAAGCGGACTCTAAGGACGGAGCATTCCGTACAGTCATCGGAACCATGGTCACTGACGCTGGTCAAGAGTGGTCTGCCGCTGTGATTAACGGTAAGTTACCAGAACGAACCCCAGCTATGGATGCTCTGCGCAGAATCCGCAATGCTGACCCTCAGTTGATTGCTGCGCTATACCCAGACCAAGCTGAGCTATTCCTGACGATGGACATGATGGACAAGCAGGGTATTGACCCTCAGGTTATTCTTGa                                                                     <  1:294073/301‑1 (MQ=255)`  
                                                                                                                                                                                                                                          `gTATGGACATTCCAGACGGTGCCAAGGATGCTATGAAGTTGAAGTACCTTCAAGCGGACTCTAAGGGCGGAGCATTCCGTACAGCCATCGGAACCATGGTCACTGACGCTGGTCAAGAGTTGTCTGCCGCTGTGATTAACGGTAAGTTACCAGAACGAACCCCAGCTATGGATGCTCTGCGCAGAATCCGCAATGCTGACCCTCAGTTGATTTCTGCGCTATACCCAGACCAAGCTGAGCTATTCCTGACGATGGACATGATGGACAAGCAGGGTATTGACCCTCAGGTTATTCtctgtct                                                                  >  1:257989/1‑295 (MQ=255)`  
                                                                                                                                                                                                                                                   `ttCCAGACGGTGCCAAGGATGCTATGAAGTTGAAGTACCTTCAAGCTGACTCTAATGGCGGAGCATTCCGTACAGCCATCGGAACCATGGTCACTGACGCTGGTCAAGAGTGGTCTGCCGCTGTGATTAACGGTAAGTTACCAGAACGAACCCCAGCTATGGATGCTCTGCGCAGAATCCGCAATGCTGACCCTCAGTTGATTGCTGCGCTATACCCAGACCAAGCTGAGCTATTCCTGACGATGGACATGATGGACAAGCAGGGTATTGACCCTCAGGTTATTCTTGATGCCGACCgact                                                         >  1:188341/1‑301 (MQ=255)`  
                                                                                                                                                                                                                                                         `ttgtataagagacagCTATGAAGTTGAAGTACCTTCAAGCGGACTCAAAGGGCGGAGCATTCCGTACAGCCATCGGAACCATGGTCACTTACGCTGGTCAAGAGTGGTCTGCCGCTGTGATTAACGGTAAGTTACCAGAACGAACCCCAGCTATGGATGCTCTGCGCAGAATCCGCAATGCTGACCCTCAGTTGATTGCTGCGCTATACCCAGACCAAGCTGAGCTATTCCTGACGATGGACATGATGGACAAGCAGGGTATTGACCCTCAGGTTATTCTTGATGCCGACCGACTGACTGt                                                   <  1:98016/287‑1 (MQ=255)`  
                                                                                                                                                                                                                                                            `gTGCCAAGGATGCTATGAAGTTGAAGTACCTTCAAGCGGACTCTAAGGACGGAGCATTCCGTACAGCCATCGGAACCATGGTCACTGACGCTGGTCAAGAGTGGTCTGCCGCTGTGATTAACGGTAAGTTACCAGAACGAACCCCAGCTATGGATGCTCTGCGCAGAATCCGCAATGCTGACCCTCAGTTGATTGCTGCGCTATACCCAGACCAAGCTGAGCTATTCCTGACGATGGACATGATGGACAAGCAGGGTATTGACCCTCAGGTTATTCTTGATGCCGACCGACTGACTGTTaa                                                >  1:1448/1‑301 (MQ=255)`  
                                                                                                                                                                                                                                                             `ggCCAAGGATGCTATGAAGTTGAAGTACCTTCAAGCGGACTCTAAGGGCGGAGCATTCCGTACAGCCATCGGAACCATGGTCACTGACGCTGGTCAAGAGTGGTCTGCCGCTGTGATTAACGGTAAGTTACCAGAACGAACCCCAGCTATGGATGCTCTGCGCAGAATCCGCAATGCTGACCCTCAGTTGATTGCTGCGCTATACCCAGACCAAGCTGAGCTATTCCTGACGATGGACATGATGGACAAGCAGGGTATTGACCCTCAGGTTATTCTTGATGCCGACCGACTGACTGTTAAg                                               <  1:238260/300‑1 (MQ=255)`  
                                                                                                                                                                                                                                                              `gCCAAGGATGCTATGAAGTTGAAGTACCTTCAAGCGTACTCTAAGGACGGAGCATTCCGTACAGCCATCGGAACCATGGTCACTGACGCTGGTCAAGAGTGGTCTGCCGCTGTGATTAACGGTAAGTTACCAGAATGAACCCCAGCTATGGATGCTCTGCGCAGAATCCGCAATGCTGACCCTCAGTTGATTGCTGCGCTATACCCAGACCAAGCTGAGCTATTCCTGACGATGGACATGATGGACAAGCAGGGTATTGACCCTCAGGTTATTCTTGATGCCGACCGACTGACTGTTAAGc                                              <  1:160151/301‑1 (MQ=255)`  
                                                                                                                                                                                                                                                              `gCCAAGGATGCTATGAAGTTGAAGTACCTTCAAGCGGACTCTAAGGGCGGAGCATTCCGTACAGCCATCGGAACCATGGTCACTGACGCTGGTCAAGAGTGGTCTGCCGCTGTGATTAACGGTAAGTTACCAGAACGAACACCAGCTATGGATGCTCTGCGCAGAATCCGCAATGCTGACCCTCAGTTGATTGCTGCGCTATACCCAGACCAAGCTGAGCTATTCCTGACGATGGACATGATGGACAAGCAGGGTATTGACCCTCAGGTTATTCTTGATGCCGACCGACTGACTGTTAAGc                                              <  1:263610/301‑1 (MQ=255)`  
                                                                                                                                                                                                                                                              `gCCAAGGATGCTATGAAATTGAAGTACCTTCAAGCGGGCTCTAAGGGCGGAGCTGTCCGTACAGCCATCGGAACCATGGTCACTGACGCTGGTCAAGCGTGGTCTGCCGCTGTGATTAACGGTAAGTTACCAGAACGAACCCCAGCAAGGGATGCTCTGCGCAGAATCCGCAATGCTGACCCTCAGTTGATTGCTGCGCTATACCAAGACCAAGCTGAGCTATTCCTGACGATGGACATGATGGACAAGCAGGGTATTGACCCTCAGGTTATTCTTGATGCCGACCGACTGACTGTTAAGc                                              <  1:300933/301‑1 (MQ=255)`  
                                                                                                                                                                                                                                                               `ccAAGGATGCTATGAAGTTGAAGTACCTTCAAGCGGACTCTAAGGGCGGAGCATTCCGTACAGCCATCGGAACCATGGTCACTGACGCTGGTCAAGAGTGGTCTGCCGCTGTGATTAACGGTAAGTTACCAGAACGAACCCCAGCTATGGATGCTCTGCGCAGAATCCGCAATGCTGACCATCAGTTGATTGCTGCGCTATACCCAGACCAAGCTGAGCTATTCCTGACGATGGACATGATGGACAAGCAGGGTATTGACCCTCAGGTTATTCTTGATGCCGACCGACTGACTGtctgtct                                             >  1:7834/1‑295 (MQ=255)`  
                                                                                                                                                                                                                                                                   `ggATGCTATGAAGTTGAAGTACCTTCAAGCGGACTCTAAGGGCGGAGCATTCCGTACAGCCATCGGAACCATGGTCACTGACGCTGGTCAAGAGTGGTCTGCCGCTGTGATTAACGGTAAGTTACCAGAACGAACCCCAGCTATGGATGCTCTGCGCAGAATCCGCAATGCTGACCCTCAGTTGATTGCTGCGCTATACCCAGACCAAGCTGAGCTATTCCTGACGATGGACATGATGGACAAGCAGGGTATTGACCCTCAGGTTATTCTTGATGCCGACCGACTGACTGTTAAGcctgtc                                         >  1:117238/1‑296 (MQ=255)`  
                                                                                                                                                                                                                                                                        `cTATGAAGTTGAAGTACCTTCAAGCGGACTCGAAGGACGGAGCATTCCGTACAGCCATCGGAACCATGGTCACTGACGCTGGTCAAGAGTGGTCTGCCGCTGTGATTAACGGTAAGTTACCAGAACGAACCCCAGCTATGGATGCTCTGCGCAGAATCCGCAATGCTGACCCTCAGTTGATTGCTGCGCTATACCCAGACCAAGCTGAGCTATTCCTGACGATGGACATGATGGACAAGCAGGGTATTGACCCTCAGGTTATTCTTGATGCCGACCGACTGACTGTTAAGCGGTCAAAaga                                    <  1:335329/301‑1 (MQ=255)`  
                                                                                                                                                                                                                                                                               `gttgaagtACCTTCAAGTGGACTCTAAGGACGGAGAATTCCGTACAGCCATCGGAACCATGGTCACTGACGCTGGTCAAGAGTGGTCTGCCGCTGTGATTAACGGTAAGTTACCAGAACGAACCCCAGCTATGGATGCTCTGCGCAGAATCCGCAATGCTGACCCTAAGTTGATTGCTGCGCTATACCCAGACCAAGCTGAGCTATTCCTGACGATGGACATGATGGACAAGCAGGGTATTGACCCTCAGGTTATTCTTGATGCCGACCGACTGACTGTTAAGCGGTCCAAAGAGCAACGc                             <  1:105628/301‑1 (MQ=255)`  
                                                                                                                                                                                                                                                                                `ttgaagtACCTTCAAGCGGACTCTAAGGGCGGAGCATTCCGTACAGCCATCGGAACCATGGTCACTGACGCTGGTCAAGAGTGGTCTGCCGCTGTGATTAACGGTAAGTTACCAGAACGAACCCCAGCTATGGATGCTCTGCGCAGAATCCGCAATGCTGACCCTCAGTTGATTGCTGCGCTATACCCAGACCAAGCTGAGCTATTCCTGACGATGGACATGATGGACAAGCAGGGTATTGACTCTCAGGTTATTCTTGATGCCGACCGACTGGCTGTTAAGCGGTCCAAAGAGCAACgag                            >  1:72781/1‑299 (MQ=255)`  
                                                                                                                                                                                                                                                                                 `tgaagtACCTTCAAGCGGACTCTAAGGGCGTAGCATTCCGTACAGCCATTGGAACCATGGTCACTGACGCTGGTCAAGAGTGGTCTGCCGCTGTGATTAACGGTAAGTTACCAGAACGAACCCCAGCTATGGATGCTCTGCGCAGAATCCGCAATGCTGACCCTCAGTTGATTGCTGCGCTATACCCAGACCAAGCTGAGCTATTCCTGACGATGGACATGATGGACAAGCAGGGTATTGACCCTCAGGTTATTCTTGATGCCGACCGACTGACTGTTAAGCGGTCCAAAGAGCAACGCtt                           <  1:338876/301‑1 (MQ=255)`  
                                                                                                                                                                                                                                                                                      `tACCTTCAAGCGGACTCTAAGGGCGGAGCATTCCGTACAGCCATCGGAACCATGGTCACTGACGCTGGTCAAGAGTGGTCTGCCGCTGTGATTAACGGTAAGTTACCAGAACGAACCCCAGCTATGGATGCTCTGCGCAGAATCCGCAATGCTGACCCTCAGTTGATTGCTGCGCTATACCCAGACCAAGCTGAGCTATTCCTGACGATGGACATGATGGACAAGCAGGGTATTGACCCTCAGGTTATTCTTGATGCCGACCGACTGACTGTTAAGCGGTCCAAAGAGCAACGCTTTGAgg                      <  1:277237/301‑1 (MQ=255)`  
                                                                                                                                                                                                                                                                                       `aCCTTCAAGCGGACTCTAAGGGCGGAGCATTCCGTACAGCCATCGGAACCATGGTCACTGACGCTGGTAAAGAGTGGTCTGCCGCTGTGATTAACGGTAAGTTACCAGAACGAACCCCAGCTATGGATGCTCTGAGCAGAATCCGCAATGCTGACCCTCAGTTGATTGCTGCGCTATACCCAGACCAAGCTGAGCTATTACTGACGATGGACATGATGGACAAGCAGGGTATTGACTCTCAGGTTATTCTTGATGCCGCCCGACTGACGGTTAAGAGGTCCAAAGAGCAACGCTTTGAggg                     >  1:36063/1‑300 (MQ=255)`  
                                                                                                                                                                                                                                                                                                 `cagctctAAGGGCGGAGCATTCCGTACAGCCATCGGAACCATGGTCACTGACGCTGGTCAAGAGTGGTCTGCCGCTGTGATTAACGGTAAGTTACCAGAACGAACCCCAGCTATGGATGCTCTGCGCAGAATCCGCAATGCTGACCCTCAGTTGATTGCTGCGCTATACCCAGACCAAGCTGAGCTATTCCTGACGATGGACATGATGGACAAGCAGGGTATTGACCCTCAGGTTATTCTTGATGCCGACCGACTGACTGTTAAGCGGTCCAAAGAGCAACGCTTTGAGGATGATAAAGCa           <  1:200107/298‑1 (MQ=255)`  
                                                                                                                                                                                                                                                                                                   `aCTCTAAGGACGGAGAATTCCGTACAGCCATCGGAACCATGGTCACTGACGCTGGTCAAGAGTGGTCTGCCGCTGTGATTAACGGTAAGTTACCAGAACGAACCCCAGCTATGGATGCTCTCCGCAGAATCCGCAATGCTGACACTCAGTTGATTGCTGCGATATACCCAGACCAAGCTGCGCTATCCCTTACGATGGACATGATGGACAAGCAGGGTATTGACCCTCGGGGTATTCTTTATTCCGACCGACTGATTGTTAATCGGTCCAACGAGCACCCACTTGAGGATGAGAAAGCAtt         >  1:228498/1‑301 (MQ=255)`  
                                                                                                                                                                                                                                                                                                    `ctctAAGGTCGGAGCATTCCGTACAGCCATCGGAACCATGGTCACTGACGCTGGTCAAGAGTGGTCTGCCGCTGTGATTAACGGTAAGTTACCAGAACGAACCCCAGCTATGGATGCTCTGCGCAGAATCCGCAATGCTGACCCTCAGTTGATTGCTGCGCTATACCCAGACCAAGCTGAGCTATTCCTGACGATGGACATGATGGACAAGCAGGGTATTGACCCTCAGGTTATTCTTGATGCCGACCGACTGACTGTTAAGCGGTCCAAAGAGCAACGCTTTGAGGATGATAAAGCATTc        >  1:23441/1‑301 (MQ=255)`  
                                                                                                                                                                                                                                                                                                    `ctctAAGGGCGGAGCATTCCGTACAGCCATCGGAACCATGGTCACTGACGCTGGTCAAGAGTGGTCTGCCGCTGTGATTAACGGTAAGTTACCAGAACGAACCCCAGCTATGGATGCTCTGCGCAGAATCCGCAATGCTGACCCTCAGTTGATTGCTGCGCTATACCCAGACCAAGCTGAGCTATTCCTGACGATGGACATGATGGACAAGCAGGGTATTGACCCTCAGGTTATTCTTGATGCCGACCGACTGACTGTTAAGCGGTCCAAAGAGCAACGCTTTGAGGATGATAAAGCATTc        >  1:115120/1‑301 (MQ=255)`  
                                                                                                                                                                                                                                                                                                    `ctctAAGGACGGAGCATTCCGTACAGCCATCTGAACCATGGTCACTGACGCTGGTCAAGAGTGGTCTGCCGCTGTGATTAACGGTAAGTTACCAGAACGAACCCCAGCTATGGATGCTCTGCGCAGAATCCGCAATGCTGACCCTCAGTTGATTGCTTCGCTATACCCAGACCAAGCTGAGCTATTCCTGACGCTGGACATGATGGACAAGCAGGGTATTGACCCTCAGGTTATTCTTGATGCCGACCGACTGACTGTTAAGCGGTCCAAAGAGCAACGCTTTGAGGATGATAAAGCAttt        >  1:151393/1‑300 (MQ=255)`  
                                                                                                                                                                                                                                                                                                    `ctctAAGGACGGAGAATTCCGTACAGCCATCGGAACCATGGTCACTGACGCTGGTCAAGAGTGGTCTGCTGCTGTGATTAACGGTAAGTTACCAGAACGAACCCCAGCTATGGATGCTCTGCGCAGAATCCGCAATGCTGACCCTCAGTTGATTGCTGCGCTATACCCAGACCAAGCTGAGCTATTCCTGACGATGGACATGATGGACAAGCAGGGTATTGACCCTCAGGTTATTCTTGATGCCGACCGACTGACTGTTAAGCGGTCCAAAGAGCAACGCTTTGAGGATGATAAAGCAtct        >  1:193821/1‑299 (MQ=255)`  

                                                                                                                                                                                                                                                                                                            `|`                                                                                                                                                                                                                                                                                                            
`AGGAACAAGTTCAGAATCAGATGAACGCATGGACGAAAGCTCAGGCCAAGGCTCTGGACGATTCCATGAAGTCAATGAACAAACTTGACGTAATCGACAAGCAATTCCAGAAGCGAATCAACGGTGAGTGGGTCTCAACGGATTTTAAGGATATGCCAGTCAACGAGAACACTGGTGAGTTCAAGCATAGCGATATGGTTAACTACGCCAATAAGAAGCTCGCTGAGATTGACAGTATGGACATTCCAGACGGTGCCAAGGATGCTATGAAGTTGAAGTACCTTCAAGCGGACTCTAAGGACGGAGCATTCCGTACAGCCATCGGAACCATGGTCACTGACGCTGGTCAAGAGTGGTCTGCCGCTGTGATTAACGGTAAGTTACCAGAACGAACCCCAGCTATGGATGCTCTGCGCAGAATCCGCAATGCTGACCCTCAGTTGATTGCTGCGCTATACCCAGACCAAGCTGAGCTATTCCTGACGATGGACATGATGGACAAGCAGGGTATTGACCCTCAGGTTATTCTTGATGCCGACCGACTGACTGTTAAGCGGTCCAAAGAGCAACGCTTTGAGGATGATAAAGCATTCGAGTCT  >  NC_001604.1/29391‑29989`

  

Alignment Legend

Aligned base mismatch/match (shaded by quality score): `ATCG/ATCG` `< 3 ≤` `ATCG/ATCG` `< 11 ≤` `ATCG/ATCG` `< 24 ≤` `ATCG/ATCG` `< 35 ≤` `ATCG/ATCG` `< 38 ≤` `ATCG/ATCG`

Unaligned base: `atcg`    Masked matching base: `atcg`    Alignment gap: `‑`    Deleted base: `‑`
